# Supplementary material for: Could optical coherence tomography detect monosodium urate crystal deposition at artery walls? An exploratory, phantom-based study
Source: PLoS One. 2025 Apr 3;20(4):e0320742. doi: 10.1371/journal.pone.0320742 (PMC11967950; doi:10.1371/journal.pone.0320742)
Supplement: S2 Table — CI: confidence interval; CPP: calcium pyrophosphate; MSU: monosodium urate; NC: not calculable; NLR: negative likelihood ratio; NPV: negative predictive value; PLR: positive likelihood ratio; PPV: positive predictive value; Sn: sensitivity; Sp: specificity. (DOCX) [file pone.0320742.s002.docx]

**Could optical coherence tomography detect monosodium urate crystal deposition at artery walls? An exploratory, phantom-based study.**

*Supplementary material*

**S2 Table**. Identification of the exploratory definitions across the vessel phantoms examined, comparing between those injected with monosodium urate crystals and those injected with saline acting as controls.

|  | **Definition #1: *lesion with high attenuation*** | |  |  |  |  |  |  |  |
| --- | --- | --- | --- | --- | --- | --- | --- | --- | --- |
|  | Yes | No | Total | Sn, % (95%CI) | Sp, % (95%CI) | PPV, % (95%CI) | NPV, % (95%CI) | PLR (95%CI) | NLR (95%CI) |
| **Whole sample** |  |  |  |  |  |  |  |  |  |
| · MSU crystals | 3 | 5 | 8 | 37.5 (0.0-77.3) | 87.5 (58.3-100.0) | 75.0 (20.1-100.0) | 58.3 (26.3-90.4) | 3.0 (0.4-23.1) | 0.7 (0.4-1.3) |
| · Saline | 1 | 7 | 8 |  |  |  |  |  |  |
| · Total | 4 | 12 | 16 |  |  |  |  |  |  |
| **Compliant phantom** |  |  |  |  |  |  |  |  |  |
| · MSU crystals | 1 | 3 | 4 | 25.0 (0.0-79.9) | 75.0 (20.1-100.0) | 50.0 (0.0-100.0) | 50.0 (1.7-98.3) | 1.0 (0.1-11.0) | 1.0 (0.5-2.2) |
| · Saline | 1 | 3 | 4 |  |  |  |  |  |  |
| · Total | 2 | 6 | 8 |  |  |  |  |  |  |
| **Semirigid phantom** |  |  |  |  |  |  |  |  |  |
| · MSU crystals | 2 | 2 | 4 | 50.0 (0.0-100.0) | 100.0 (87.5-100.0) | 100.0 (75.0-100.0) | 66.7 (20.6-100.0) | NC | 0.5 (0.2-1.3) |
| · Saline | 0 | 4 | 4 |  |  |  |  |  |  |
| · Total | 2 | 6 | 8 |  |  |  |  |  |  |
|  | **Definition #2: *Linear lesion with high attenuation*** | |  |  |  |  |  |  |  |
|  | Yes | No |  |  |  |  |  |  |  |
| **Whole sample** |  |  |  |  |  |  |  |  |  |
| · MSU crystals | 2 | 6 | 8 | 25.0 (7.2-59.1) | 100.0 (67.6-100.0) | 25.0 (7.2-59.1) | 100.0 (67.6-100.0) | NC | 75.0 (50.3-1.12) |
| · Saline | 0 | 8 | 8 |  |  |  |  |  |  |
| · Total | 2 | 14 | 16 |  |  |  |  |  |  |
| **Compliant phantom** |  |  |  |  |  |  |  |  |  |
| · MSU crystals | 0 | 4 | 4 | NC | 50.0 (21.5-78.9) | 0.0 (0.0-48.9) | 100.0 (51.1-100.0) | NC | NC |
| · Saline | 0 | 4 | 4 |  |  |  |  |  |  |
| · Total | 0 | 8 | 8 |  |  |  |  |  |  |
| **Semirigid phantom** |  |  |  |  |  |  |  |  |  |
| · MSU crystals | 2 | 2 | 4 | 50.0 (15.0-85.0) | 100.0 (51.0-100.0) | 50.0 (15.0-85.0) | 100.0 (51.1-100.0) | NC | 0.5 (0.2-1.3) |
| · Saline | 0 | 4 | 4 |  |  |  |  |  |  |
| · Total | 2 | 6 | 8 |  |  |  |  |  |  |

CI: confidence interval; CPP: calcium pyrophosphate; MSU: monosodium urate; NC: not calculable; NLR: negative likelihood ratio; NPV: negative predictive value; PLR: positive likelihood ratio; PPV: positive predictive value; Sn: sensitivity; Sp: specificity.

**Table S2**. Identification of the exploratory definitions across the vessel phantoms examined, comparing between those injected with monosodium urate crystals and those injected with calcium pyrophosphate crystals acting as controls.

|  | **Definition #1: *lesion with high attenuation*** | |  |  |  |  |  |  |  |
| --- | --- | --- | --- | --- | --- | --- | --- | --- | --- |
|  | Yes | No | Total | Sn, % (95%CI) | Sp, % (95%CI) | PPV, % (95%CI) | NPV, % (95%CI) | PLR (95%CI) | NLR (95%CI) |
| **Whole sample** |  |  |  |  |  |  |  |  |  |
| · MSU crystals | 3 | 5 | 8 | 37.5 (0.0-77.3) | 75.0 (38.7-100.0) | 60.0 (7.1-100.0) | 54.6 (20.6-88.5) | 1.5 (0.3-6.7) | 0.8 (0.4-1.6) |
| · CPP crystals | 2 | 6 | 8 |  |  |  |  |  |  |
| · Total | 5 | 11 | 16 |  |  |  |  |  |  |
| **Compliant phantom** |  |  |  |  |  |  |  |  |  |
| · MSU crystals | 1 | 3 | 4 | 25.0 (0.0-79.9) | 75.0 (20.1-100.0) | 50.0 (0.0-100.0) | 50.0 (1.7-98.3) | 1.0 (0.1-11.0) | 1.0 (0.5-2.2) |
| · CPP crystals | 1 | 3 | 4 |  |  |  |  |  |  |
| · Total | 2 | 6 | 8 |  |  |  |  |  |  |
| **Semirigid phantom** |  |  |  |  |  |  |  |  |  |
| · MSU crystals | 2 | 2 | 4 | 50.0 (0.0-100.0) | 75.0 (20.1-100.0) | 66.7 (0.0-100.0) | 60.0 (7.1-100.0) | 2.0 (0.3-14.2) | 0.7 (0.2-2.1) |
| · CPP crystals | 1 | 3 | 4 |  |  |  |  |  |  |
| · Total | 3 | 5 | 8 |  |  |  |  |  |  |
|  | **Definition #2: *Linear lesion with high attenuation*** | |  |  |  |  |  |  |  |
|  | Yes | No |  |  |  |  |  |  |  |
| **Whole sample** |  |  |  |  |  |  |  |  |  |
| · MSU crystals | 2 | 6 | 8 | 25.0 (7.2-59.1) | 100.0 (67.6-100.0) | 25.0 (7.2-59.1) | 100.0 (67.6-100.0) | NC | 75.0 (50.3-1.12) |
| · CPP crystals | 0 | 8 | 8 |  |  |  |  |  |  |
| · Total | 2 | 14 | 16 |  |  |  |  |  |  |
| **Compliant phantom** |  |  |  |  |  |  |  |  |  |
| · MSU crystals | 0 | 4 | 4 | NC | 50.0 (21.5-78.9) | 0.0 (0.0-48.9) | 100.0 (51.1-100.0) | NC | NC |
| · CPP crystals | 0 | 4 | 4 |  |  |  |  |  |  |
| · Total | 0 | 8 | 8 |  |  |  |  |  |  |
| **Semirigid phantom** |  |  |  |  |  |  |  |  |  |
| · MSU crystals | 2 | 2 | 4 | 50.0 (15.0-85.0) | 100.0 (51.0-100.0) | 50.0 (15.0-85.0) | 100.0 (51.1-100.0) | NC | 0.5 (0.2-1.3) |
| · CPP crystals | 0 | 4 | 4 |  |  |  |  |  |  |
| · Total | 2 | 6 | 8 |  |  |  |  |  |  |
